# The signaling protein GIV/Girdin mediates the Nephrin-dependent insulin secretion of pancreatic islet β cells in response to high glucose

**DOI:** 10.1016/j.jbc.2023.103045

**Published:** 2023-02-21

**Authors:** Hao Wang, Ying-Chao Yuan, Cong Chang, Tetsuro Izumi, Hong-Hui Wang, Jin-Kui Yang

**Affiliations:** 1 Beijing Key Laboratory of Diabetes Research and Care, Beijing Diabetes Institute, Beijing Tongren Hospital, Capital Medical University, Beijing, China; 2 College of Biology, Hunan University, Changsha, Hunan, China; 3 Hunan Food and Drug Vocational College, Changsha, Hunan, China; 4 Laboratory of Molecular Endocrinology and Metabolism, Department of Molecular Medicine, Institute for Molecular and Cellular Regulation, Gunma University, Maebashi, Gunma, Japan

**Keywords:** GIV, tyrosine phosphorylation, Nephrin, actin remodeling, insulin exocytosis

## Abstract

Glucose-stimulated insulin secretion of pancreatic β cells is essential in maintaining glucose homeostasis. Recent evidence suggests that the Nephrin-mediated intercellular junction between β cells is implicated in the regulation of insulin secretion. However, the underlying mechanisms are only partially characterized. Herein we report that GIV is a signaling mediator coordinating glucose-stimulated Nephrin phosphorylation and endocytosis with insulin secretion. We demonstrate that GIV is expressed in mouse islets and cultured β cells. The loss of function study suggests that GIV is essential for the second phase of glucose-stimulated insulin secretion. Next, we demonstrate that GIV mediates the high glucose-stimulated tyrosine phosphorylation of GIV and Nephrin by recruiting Src kinase, which leads to the endocytosis of Nephrin. Subsequently, the glucose-induced GIV/Nephrin/Src signaling events trigger downstream Akt phosphorylation, which activates Rac1-mediated cytoskeleton reorganization, allowing insulin secretory granules to access the plasma membrane for the second-phase secretion. Finally, we found that GIV is downregulated in the islets isolated from diabetic mice, and rescue of GIV ameliorates the β-cell dysfunction to restore the glucose-stimulated insulin secretion. We conclude that the GIV/Nephrin/Akt signaling axis is vital to regulate glucose-stimulated insulin secretion. This mechanism might be further targeted for therapeutic intervention of diabetic mellitus.

The pancreatic islet β cells dynamically regulate insulin secretion to maintain glucose homeostasis (1). The dysfunction of islet cells leads to hyperglycemia in type I and type II diabetes mellitus, the most common chronic disease affecting over 415 million people globally (2). In physiological conditions, the rise in blood glucose levels is rapidly countered by insulin secretion from pancreatic islet β cells (3). Subsequently, the released insulin can promote the uptake and storage of blood glucose in peripheral tissues, *e.g.*, the liver, adipose tissue, and skeletal muscle (4). The function of β cells for glucose-stimulated insulin secretion should be tightly regulated to ensure that a suitable amount of insulin is released. The molecular mechanism of glucose-stimulated insulin secretion remains complex, is not fully understood, and is currently a major research focus in the literature. Naturally, the process of glucose-stimulated insulin secretion of pancreatic islet β cells is biphasic (3). In the first-phase secretion, a releasable pool of insulin secretory granules localizes close to the plasma membrane, and elevated glucose triggers the calcium influx to initiate the fusion of a small number of insulin-containing granules predocked at the plasma membrane; this occurs very quickly, typically in several minutes. In contrast, the second phase of insulin secretion is a more sustained process that lasts for hours under persisting glucose stimulation (5). Under resting-state conditions, β cells have a dense web of cortical filamentous actin (F-actin) beneath the cell membrane to block the access of insulin granules to the cell periphery. In response to the elevated glucose level, β cells transduce the signaling to initiate the F-actin remodeling, which allows the movement of insulin granules to translocate and fuse with the plasma membrane for insulin release (5). However, it remains unclear how the β cells could sense the elevated glucose and transmit proper intracellular signaling to tune insulin secretion.

Recent evidence has suggested that the highly regulated intercellular junction between β cells *via* their cell surface receptors might be an important glucose-sensory interface for the proper regulation of insulin secretion (6, 7, 8). For instance, the adherens junction, composed of E-cadherin and N-cadherin, is important for properly regulating the insulin secretion of islet β cells. Physiological or pathological conditions altering the expression or subcellular localization of adherens junctions might significantly impact the β-cell function in insulin secretion (7, 9). Nevertheless, the signaling pathway coordination between the cell–cell junction and F-actin remodeling for insulin secretion remains largely unexplored. As a specialized component of adherens junction protein, Nephrin is an immunoglobulin-like protein that can combine with podocyte-associated membrane proteins, such as podocin, Neph proteins, and cadherin superfamily members, forming the slit diaphragm to bridge glomerular podocytes for regulated kidney filtration (10). In addition to mediating cell–cell junction, Nephrin can also function as the key signaling molecule that maintains the filtration slits and podocyte survival by recruiting phosphoinositide 3-kinase (PI3K) for the subsequent activation of prosurvival Akt signaling (10, 11, 12). Interestingly, Nephrin is also expressed in other tissues, such as the brain and pancreas, and it has been identified that Nephrin localizes in the cell–cell junction of β cells (13). Nephrin has been recently reported to facilitate glucose-stimulated insulin release by pancreatic beta cells through dynamin-dependent Nephrin phosphorylation and endocytosis (11). The mice with Nephrin pancreatic β cell–specific deletion have an impaired glucose sensing function of pancreatic β cells (14). However, the signaling machinery mediating the Nephrin function on the cell surface to intracellular signaling pathway and F-actin remodeling for insulin secretion in islet β cells remains uncharacterized.

GIV protein (also known as APE, Girdin, and HkRP1; gene, CCDC88A) has recently emerged as a multidomain signaling regulator to transduce the ligand-induced receptor activation at the plasma membrane and relay intracellular signaling pathways, *e.g.*, PI3K-Akt, PKA/CREB, mTOR (15, 16, 17). More specifically, GIV contains a unique Gα-binding and -activating (GBA) motif conferring guanine nucleotide exchange factor (GEF) activity on Gαi subunits to activate trimeric G proteins (18, 19). In response to receptor tyrosine kinases and GPCRs stimulation, GIV-mediated G protein activation dissociates Gβγ subunits, which enhances the PI3K-Akt signaling (20). The role of GIV in transmitting receptor signaling to promote the PI3K-Akt signaling pathway has been established in distinct cellular behaviors in various physiological and pathological conditions, such as liver fibrosis (21), tumor metastasis (22, 23), and kidney podocytes early injury (24). However, the expression of GIV in pancreatic islets and its potential role in insulin secretion have yet to be investigated.

In this study, we hypothesize that GIV may be a critical signaling modulator of Nephrin phosphorylation and endocytosis in response to extracellular high glucose concentration to regulate glucose-stimulated insulin secretion. For the first time, our work verifies the expression and subcellular localization of GIV at the cell–cell junctions of the mouse islet and islet β cells. We demonstrate that GIV can combine with Nephrin and recruit Src kinase to induce tyrosine phosphorylation of both proteins to transmit PI3K/Akt signaling. Consequently, the GIV/Nephrin/Akt axis facilitates the high glucose–induced endocytosis of Nephrin and the F-actin reorganization to promote insulin secretion. Finally, the levels of GIV and Nephrin are downregulated in diabetic mice, and the adenovirus-mediated overexpression of GIV could rescue the function of β cells in glucose-stimulated insulin secretion. Our study reveals the role of GIV in insulin secretion and identifies novel signaling machinery regulating glucose-stimulated insulin secretion, which may be a potential target for the therapy of diabetic mellitus.

## Results

### GIV is expressed in pancreatic β cells

**Figure 1:**
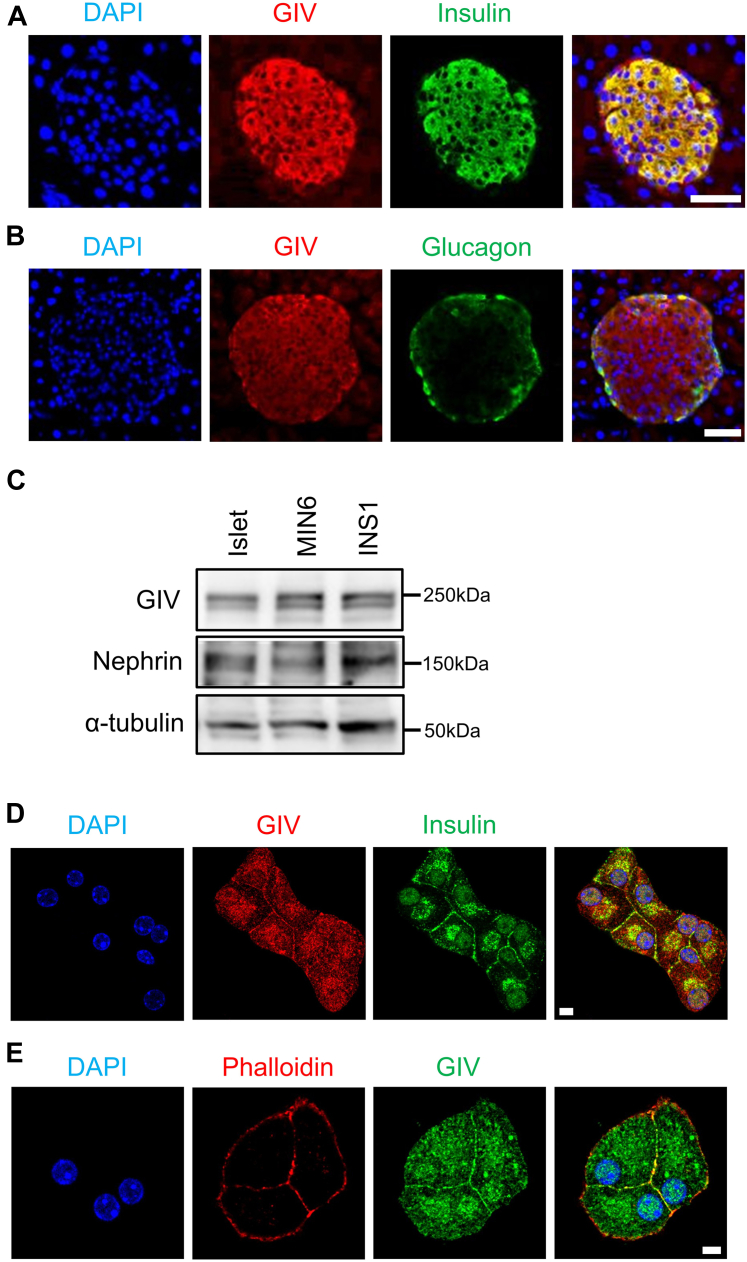
**GIV is expressed in pancreatic islets and β cells.***A* and *B*, immunofluorescence confocal images for GIV and insulin (*A*) or GIV and glucagon (*B*) in mouse pancreatic slices were shown. The scale bar represents 50 μm. *C*, Western blotting analysis for GIV and Nephrin in mouse islets and β cells. *D* and *E*, immunofluorescence staining of GIV and insulin (*D*) or GIV and Phalloidin (*E*) in MIN6 cells. The scale bar represents 5 μm. All the experiments were repeated at least three times.

We initially explored the tissue distribution of GIV. The results reveal that both mRNA and protein of GIV were widely expressed in many organs. Specifically, GIV was highly expressed in the brain and moderately in other tissues investigated, including endocrine tissues such as the adrenal gland, pancreas, and pituitary gland (Fig. S1, *A* and *B*). We investigated GIV expression in a normal murine pancreas. Immunostaining revealed that GIV was produced in both α and β cells in the islets of control mice (Fig. 1, *A* and *B*). GIV expression in murine islets, murine insulinoma cells, and rat insulinoma cells was confirmed by Western blot (Fig. 1*C*) and quantitative PCR (Fig. S1*B*). We then investigated the intracellular localization of GIV in β cells. Double-immunostaining analyses indicated that GIV was partially colocalized with insulin granules in murine β cells (Fig. 1*D*). Furthermore, we found that GIV was colocalized with F-actin close to the cell periphery (Fig. 1*E*), similar to the distribution of GIV in cos7 cells. These results indicate that GIV was expressed in pancreatic β cells, primarily localized on the plasma membrane.

### GIV/Girdin regulates glucose-stimulated insulin secretion in MIN6 cells and murine islets

**Figure 2:**
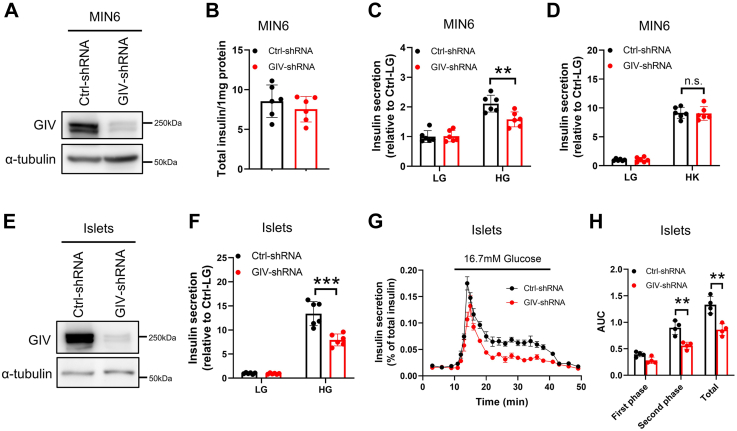
**GIV regulated glucose-induced insulin secretion in MIN6 cells and mouse islets.** MIN6 cells were infected with shRNA for 2 days. *A*, GIV protein levels were checked by immunoblotting (n = 3). *B*–*D*, the effect of GIV shRNA on total insulin content (*B*), glucose-induced insulin secretion (*C*), and KCl-induced insulin secretion in MIN6 cells (*D*) (n = 6). Islets from C57BL/6J mice (12–16 weeks old) were infected with shRNA for 2 days. *E*, GIV protein levels were checked by immunoblotting (n = 3). *F*, the effect of GIV shRNA on glucose-induced insulin secretion in mouse islets (n = 6). *G*, shRNA-treated mouse islets were perifused with Krebs–Ringer bicarbonate buffer containing 16.7 mM glucose for 30 min, followed by the standard buffer (2.8 mM glucose) for 10 min. Insulin secretion was normalized to the total insulin content. *H*, first phase (0–7 min), second phase (8–30 min), and total insulin secretion were calculated as the area under the curve (AUC) (n = 4). The statistical significance of differences between means was assessed by the Student’s *t* test. ∗∗*p* < 0.01; ∗∗∗*p* < 0.001; n.s. means not significant.

To further characterize the role of GIV in β-cell function, MIN6 cells were infected with lentivirus coding short hairpin RNA (shRNA) of GIV for downregulating the endogenous expression level of GIV, and about 66.2% of GIV was reduced after knockdown (Fig. 2*A*). The depletion of GIV expression does not affect the total insulin content (Fig. 2*B*). Glucose-stimulated insulin secretion (GSIS) assay showed that GIV depletion profoundly decreased GSIS of MIN6 cells (Fig. 2*C*). Interestingly, downregulation of GIV did not affect KCl-stimulated insulin secretion (Fig. 2*D*). To overcome the limitation of using insulinoma cells to investigate the role of GIV in insulin secretion, we applied GIV shRNA downregulation to dissociated murine islet cells. About 80.5% of GIV in shRNA-treated murine islets was robustly reduced (Fig. 2*E*). We confirmed that knockdown of GIV could decrease GSIS in murine islets (Fig. 2*F*). To directly examine the insulin secretion ability, we performed perifusion analyses in isolated islets, as described (25). GIV shRNA affected glucose-induced insulin secretion in perifused islet cells compared with nontargeting shRNA (Fig. 2*G*). The area under the curve shows that only the second phase (6–30 min) of insulin secretion was markedly reduced in GIV-diminished islets (Fig. 2*H*). These results indicate that GIV is crucial for GSIS in MIN6 cells and mouse islets, especially for the second phase.

### GIV is required for glucose-induced phosphorylation of Nephrin and Akt

**Figure 3:**
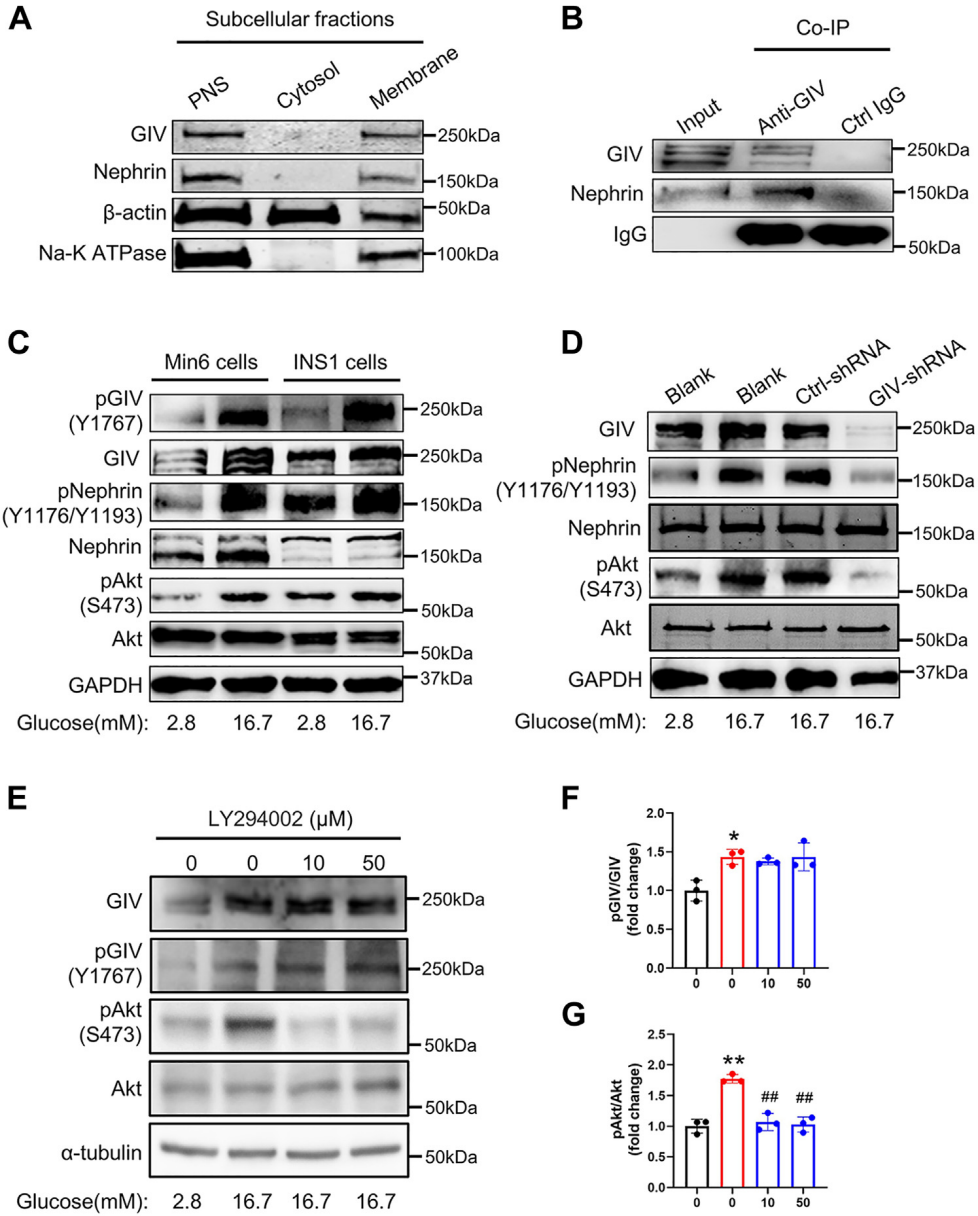
**GIV is required for glucose-induced phosphorylation of Nephrin and Akt.***A*, subcellular location of GIV in MIN6 cells. The cytosolic and crude membrane fractions were isolated by ultracentrifugation. Postnucleation supernatant (PNS) indicates the total expression of proteins, β-actin and sodium and potassium pump (Na-K-ATPase) as the marker of cytosolic and crude membrane fractions, respectively. The experiments were repeated three times. *B*, total islet protein lysates (300 mg) from wildtype mice underwent immunoprecipitation with anti-GIV antibody or control IgG. The immunoprecipitates, as well as 1:20 of the original lysates, were immunoblotted with anti-GIV and anti-Nephrin antibodies (n = 3). *C*, MIN6 and INS1 cells were stimulated with 2.8 mM or 16.7 mM glucose for 30 min, and protein expression levels were analyzed by immunoblotting with antibodies toward the indicated proteins (n = 3). *D*, effects of insulin on GIV phosphorylation in MIN6 cells. MIN6 cells were stimulated with 2.8 mM or 16.7 mM glucose for 30 min in the presence or absence of insulin; 16.7 mM glucose treated was as a positive control of GIV phosphorylation (n = 3). *E*, effects of GIV on glucose-induced GIV, Nephrin, and Akt phosphorylation in MIN6 cells. MIN6 cells were treated with or without GIV shRNA or control shRNA lentivirus for 2 days, then were stimulated with 2.8 mM or 16.7 mM glucose for 30 min (n = 3 in each group). *F*, effects of PI3K inhibitors on glucose-induced GIV, Nephrin, and Akt phosphorylation in MIN6 cells. MIN6 cells were stimulated with 2.8 mM or 16.7 mM glucose for 30 min in the presence of dimethyl sulfoxide (DMSO) (0.05%) or Ly294002 (0.5 μM or 5 μM); DMSO was treated as a control of the inhibitor (shown as 0 μM) (n = 3). *F* and *G*, the expression levels of phosphorylated proteins normalized by their total protein levels were measured by densitometry and the total protein levels were normalized by those of α-tubulin. The statistical significance of differences between means was assessed by one-way ANOVA with a Tukey’s test. ∗*p* < 0.05; ∗∗*p* < 0.01 *versus* 2.8 mM glucose, ^##^*p* < 0.01 *versus* 16.7 mM glucose with DMSO in each group).

To identify the molecular mechanism by which GIV regulates GSIS, we initially checked the subcellular expression of GIV in β cells. We isolated the cytosolic and crude membrane fractions, respectively. The results depict that GIV was almost located in the membrane fraction but not in the cytosolic fraction, similar to Nephrin, a well-known membrane protein in MIN6 cells (Fig. 3*A*). The colocalization of GIV and Nephrin shows that they might bind with each other to form a protein complex. To confirm this hypothesis, we explored the interaction between GIV and Nephrin by immunoprecipitation. The result showed that GIV could bind with Nephrin in MIN6 cells (Fig. 3*B*) and mouse pancreatic islets (Fig. S2*A*). In addition, high glucose had a negligible effect on the interaction between GIV and Nephrin compared with that in the control condition with low glucose (Fig. S2*B*), supporting that GIV constitutively binds Nephrin. Assuming that Nephrin is involved in insulin secretion caused by high glucose (13, 26), our data suggest that GIV and Nephrin might form a signaling complex to elicit intracellular downstream signaling to regulate insulin secretion. We further examined the effects of high glucose stimulation on the activation of GIV, Nephrin, and the downstream Akt protein by Western blotting. The results demonstrated that high glucose significantly increased the phosphorylation of GIV (Y1767) and Nephrin (Y1176/Y1193) in both MIN6 and INS1 cells. The phosphorylation of GIV and Nephrin has been reported to activate downstream Akt signaling (16). We next assessed the role of Y1767 phosphorylation of GIV in the glucose-triggered signaling transduction by examining the changes in the levels of phosphorylated Akt and GIV in MIN6 and INS1 cells following glucose elevation. MIN6 and INS1 cells were treated with a Krebs–Ringer bicarbonate buffer containing 2.8 mM glucose (low glucose) or 16.7 mM glucose (high glucose) for 30 min. High-glucose treatment significantly increased the phosphorylated level of GIV-Y1767 (Figs. 3*C* and S2*C*). A significant increase in the level of Nephrin phosphorylation and Akt S473 phosphorylation was also evident after high-glucose treatment (Figs 3*C* and S2*C*). To investigate the molecular relationship between GIV and Nephrin, we then examined the expression level of phosphorylated Nephrin using GIV-shRNA-infected MIN6 cells. Interestingly, we found that both phosphorylated Nephrin and Akt were remarkably reduced in GIV-depleted MIN6 cells (Figs 3*D* and S2*D*). This result indicates that the tyrosine phosphorylation of GIV at Y1767 is essential for glucose-induced Nephrin and Akt phosphorylation. Glucose-induced phosphorylation of GIV at Y1767 was not changed by pretreatment with PI3K inhibitor LY294002 (Figs. 3, *E* and *F* and S2*E*), indicating that increases in GIV-Y1767 phosphorylation were not through the PI3K/Akt pathway in MIN6 cells, the effect of LY294002 was identified by unchanged expression of Akt phosphorylation after glucose stimulation (Figs. 3*G* and S2*E*). Interestingly, the insulin stimulation in a low-glucose condition significantly enhanced the Y1767 phosphorylation of GIV as the high glucose (Fig. S3, *A*–*C*), illustrating that the glucose-induced insulin may further serve as positive feedback stimuli to contribute the GIV phosphorylation event for signal amplification. Taken together, our results indicate that GIV-Y1767 phosphorylation is an upstream event of the PI3K/Akt pathway and positively correlates with the activation of Nephrin phosphorylation and Akt phosphorylation.

### GIV phosphorylation (Y1767) mediates phosphorylation of Nephrin *via* recruitment of Src kinase

**Figure 4:**
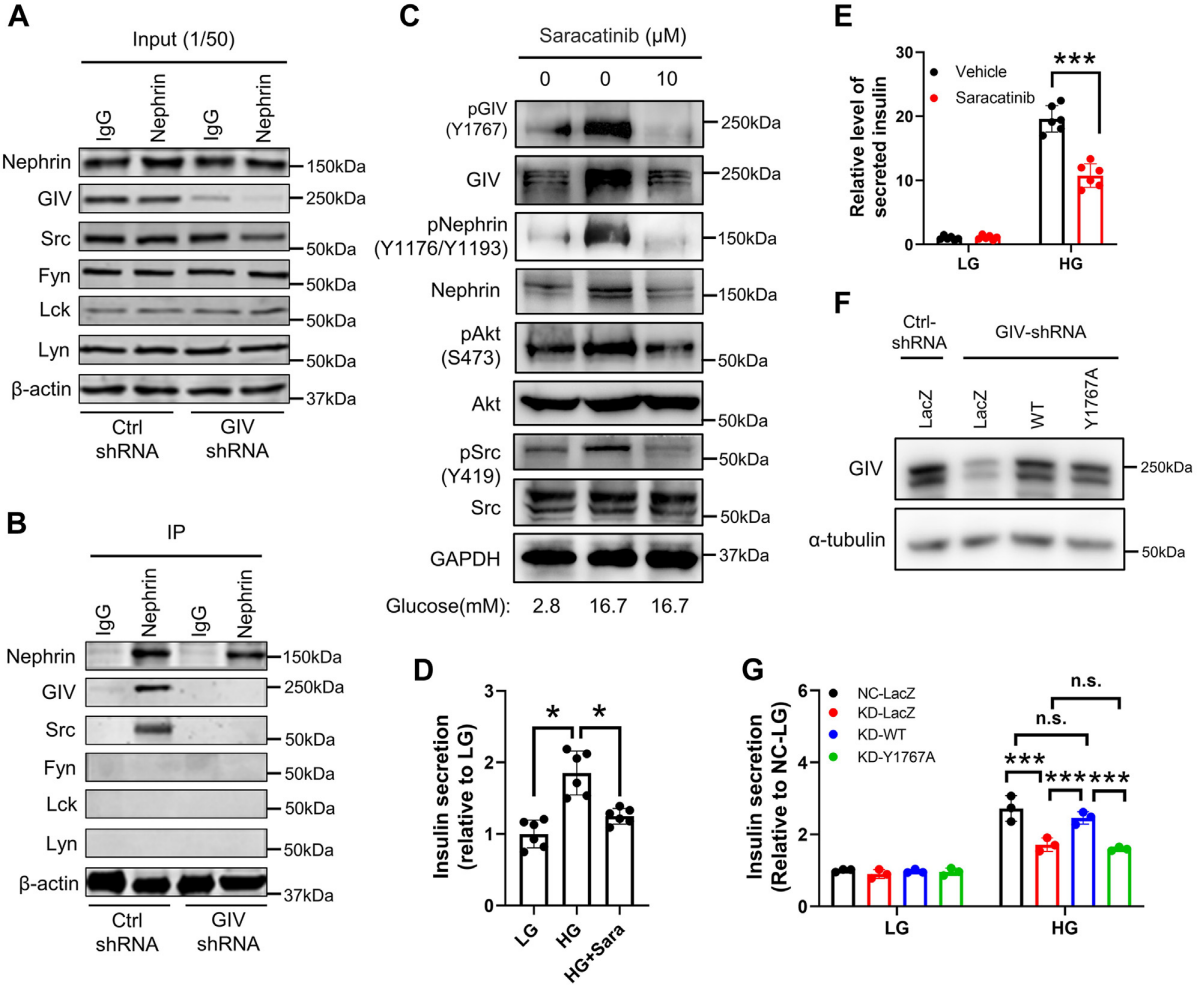
**GIV-Y1767 phosphorylation–mediated phosphorylation of Nephrin through recruitment of Src family kinase.***A* and *B*, total islet protein lysates (1 mg) from MIN6 cells underwent immunoprecipitation with anti-Nephrin antibody or control IgG. The immunoprecipitates (*B*), as well as 1:50 of the original lysates (*A*), were immunoblotted with indicated antibodies (n = 3). *C*, effects of Src family kinase on glucose-induced GIV, Nephrin, and Akt phosphorylation in MIN6 cells. MIN6 cells were stimulated with 2.8 mM or 16.7 mM glucose for 30 min in the presence of dimethyl sulfoxide (DMSO) (0.1%) or Saracatinib (10 μM). DMSO was treated as a control of Saracatinib (shown as 0 μM) (n = 3). *D* and *E*, MIN6 cells (*D*) or mouse islets (*E*) were stimulated with 2.8 mM or 16.7 mM glucose for 60 min in the presence of DMSO (0.1%) or Saracatinib (10 μM), and the amount of insulin secretion was normalized to the total insulin content followed with being normalized with the amount of insulin secretion of standard condition (n = 6). The statistical significance of differences between means was assessed by the Student’s *t* test. ∗*p* < 0.05; ∗∗∗*p* < 0.001. *F*, MIN6 cells treated with lentivirus encoding control shRNA or shRNA against GIV were infected with adenovirus encoding control LacZ, or wildtype or GIV-Y1767A mutant. The cell extracts were immunoblotted with anti-GIV and anti-a-tubulin antibodies (n = 3). *G*, MIN6 cells were subjected to glucose-stimulated insulin secretion assays as in Figure 2*C* (n = 3). The statistical significance of differences between means was assessed by one-way ANOVA with a Tukey’s test. ∗∗∗*p* < 0.001; n.s. means not significant.

Several members of the Src family kinases could induce Nephrin phosphorylation; Fyn was consistently found to be coimmunoprecipitated with Nephrin (27). A previous study has revealed that GIV lacks kinase activity and that tyrosine phosphorylation of GIV at Y1767 by the nonreceptor tyrosine kinase Src promotes activation of PI3K signaling during cell migration (28). Accordingly, we assumed Nephrin might interact with Src family kinases through GIV. To investigate what type of Src kinase family was recruited by GIV, Nephrin was immunoprecipitated from MIN6 cells treated with Ctrl-shRNA and GIV-shRNA. We found that, in GIV knockdown MIN6 cells, the expression of the indicated proteins in the cell did not change (Figs. 4*A* and S4*A*). However, Src kinase, but not Fyn and Lck, as well as Lyn kinases, was coimmunoprecipitated with Nephrin in control MIN6 cells (Fig. 4*B*). Interestingly, neither GIV nor Src was coimmunoprecipitated with Nephrin in GIV-deficient MIN6 cells, suggesting that Nephrin interacted with Src kinase through GIV (Fig. 4*B*). These findings indicate that, under normal conditions, GIV was used as a vector to recruit Src kinase interacting with Nephrin to form a complex and induced phosphorylation of Nephrin.

To further validate the role of Src in the phosphorylation of GIV and Nephrin, the effects of saracatinib, a selective Src family tyrosine kinase inhibitor (29), were examined in MIN6 cells. MIN6 cells were preincubated with vehicle (dimethyl sulfoxide) or saracatinib for 1 h before stimulation with low and high glucose. High glucose increased phosphorylation of GIV and Nephrin in addition to Akt in vehicle-treated MIN6 cells (Figs. 4*C* and S4*B*). Furthermore, phosphorylation of Src was also induced after high-glucose stimulation (Figs. 4*C* and S4*B*). However, such glucose-induced phosphorylation vanished in saracatinib-treated MIN6 cells (Figs. 4*C* and S4*B*). In addition, the glucose-induced high levels of total GIV and Nephrin were also abolished after adding Src kinase inhibitor (Fig. S4*C*), which indicated that such increment was due to a direct effect of increased phosphorylation of GIV and Nephrin. Concerning that Src kinase inhibitor erased the signaling enhancement, these findings suggest that Src kinase initially activated GIV-Y1767 phosphorylation after high glucose stimulation, subsequently promoting the GIV/Nephrin/PI3K/Akt pathway. To further evaluate the effect of Src on insulin secretion in β cells, MIN6 cells or islet cells were stimulated with low or high glucose with preincubation of saracatinib or vehicle. Saracatinib-treated MIN6 cells demonstrated significantly decreased insulin secretion compared with vehicle-treated MIN6 cells (Fig. 4*D*). Similarly, saracatinib-treated islet cells also exhibited markedly reduced insulin secretion compared with vehicle-treated islet cells (Fig. 4*E*).

Since Src kinase initially activated the Y1767 phosphorylation of GIV after high-glucose stimulation, we speculate that the phosphomodification of this tyrosine site of GIV was essential for glucose-stimulated insulin secretion. To substantiate this speculation, we performed function rescue experiments to analyze the effect of Y1767 phosphorylation of GIV on the exocytosis of insulin granules in MIN6 cells. When the wildtype or GIV-Y1767A mutant was expressed in GIV-knockdown cells as the endogenous level of GIV (Fig. 4*F*), only the wildtype rescued the decreased insulin secretion (Fig. 4*G*), supporting the importance of Y1767 phosphorylation of GIV on insulin exocytosis.

### GIV facilitates the endocytosis of Nephrin upon high-glucose stimulation

**Figure 5:**
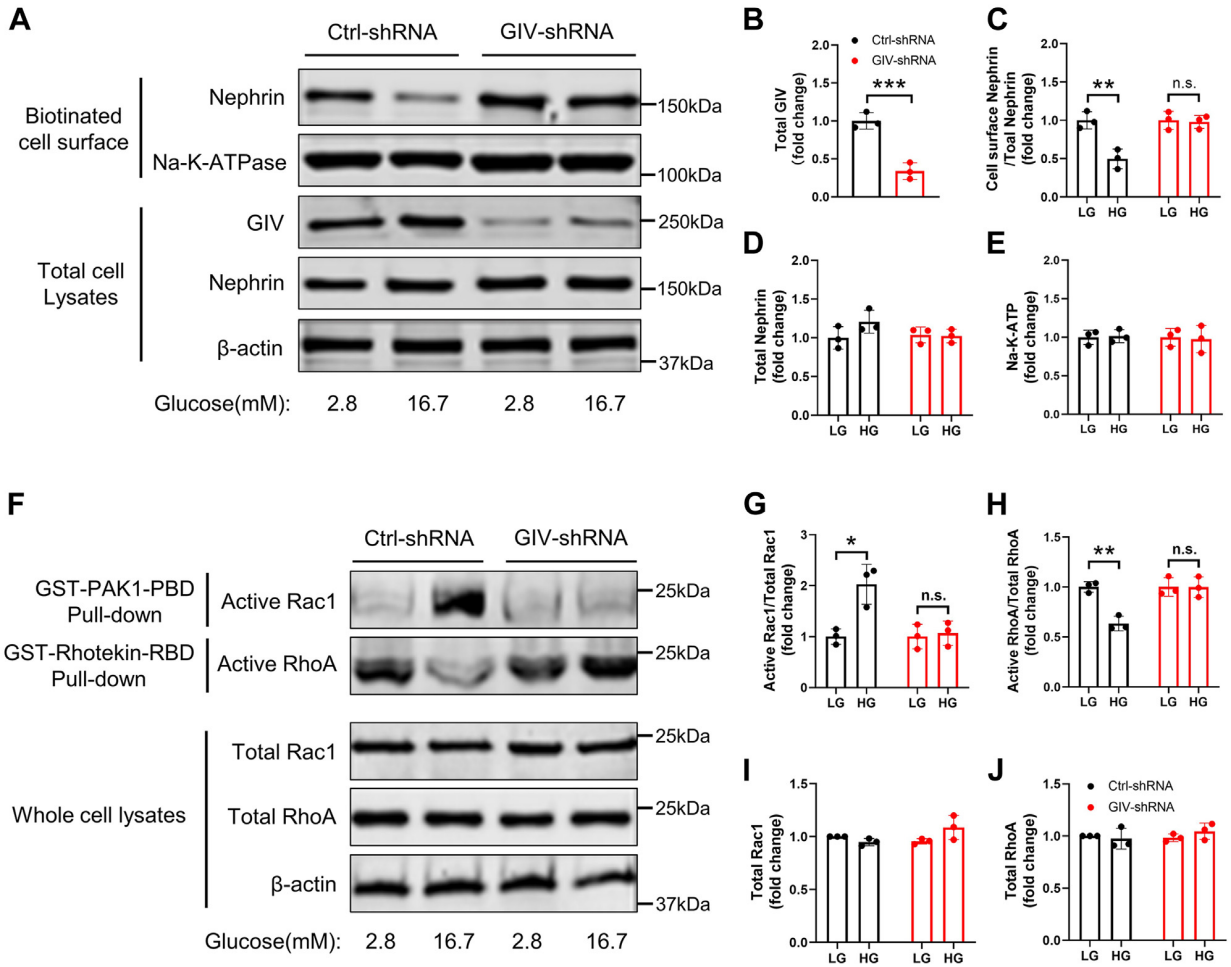
**GIV modulated F-actin remodeling through mediating endocytosis of Nephrin after high-glucose stimulation.***A*, subcellular expression of Nephrin on the plasma membrane in glucose-induced GIV downregulated MIN6 cells was checked by cell-surface biotinylation assay. *B*–*E*, the protein levels normalized by those of β-actin were measured by densitometry (n = 3). *F*, MIN6 cells were infected with shRNA for 48 h, and 2.8 mM glucose and 16.7 mM glucose were added for 30 min before cells were lysed. Cell lysates were subjected to GST-PAK1-PBD (GTP bound [active form] Rac1 interaction binding) or GST-Rhotekin-RBD (GTP bound [active form] RhoA interaction binding) pull-down followed by immunoblotting with anti-Rac1 and anti-RhoA. Normalized to total Rac1 and RhoA. *G*–*J*, the expression levels of active Rac1 and active RhoA normalized by their total protein levels were measured by densitometry, and the total protein levels of Rac1 and RhoA were normalized by those of β-actin (n = 3). The statistical significance of differences between means was assessed by the Student’s *t* test. ∗*p* < 0.05; ∗∗*p* < 0.01; ∗∗∗*p* < 0.001; n.s. means not significant.

Numerous reports reveal that Nephrin's subcellular distribution is altered by glucose concentration, as Nephrin is localized to the plasma membrane under low-glucose conditions and translocated to the cytoplasm after high-glucose stimulation (13, 26). High glucose-induced Nephrin phosphorylation accumulates more endocytosis-related proteins, such as Dynamin (11), to execute subsequent endocytosis. Since GIV regulated glucose-induced Nephrin phosphorylation, we speculate that GIV mediates the endocytosis of Nephrin after high-glucose stimulation in β cells. To further verify this possibility, we measured the expression level of Nephrin at the plasma membrane in murine β cells using a cell-surface biotinylation assay. Nephrin was detectable in plasma membrane fractions from MIN6 cells cultured in low glucose, but its intensity was markedly reduced in high glucose (Fig. 5, *A* and *C*). In contrast, the total level of Nephrin remained changed significantly (Fig. 5, *A* and *D*), suggesting that the decrease in the surface level of Nephrin could be attributed to endocytosis on the plasma membrane after high-glucose stimulation rather than the reduction in Nephrin synthesis, which is consistent with the previous studies (12, 14). To exclude the possibility of high-glucose-induced nonspecific cell surface protein endocytosis, we examined the expression level of Na-K-ATP, a cell surface protein. We found that its expression level was unaltered after high-glucose stimulation (Fig. 5, *A* and *E*). However, when analyzing the expression of Nephrin at the plasma membrane in GIV knockdown MIN6 cells (Fig. 5, *A* and *B*), Nephrin expression was explicitly present in both low and high glucose, and the high-glucose-induced Nephrin endocytosis was completely abolished (Fig. 5, *A* and *C*). These findings suggested that GIV mediated high-glucose-induced Nephrin endocytosis in murine β cells.

Nephrin endocytosis regulated PI3K/Akt-mediated actin reorganization through increasing Rac1 activity in podocytes (10). In addition, it has been reported that GIV reorganized the actin cytoskeleton in cancer cells (16). However, how GIV/Nephrin pathway regulates actin remodeling under elevated glucose in β cells remains unclear. Glucose-mediated actin remodeling in β cells highly depends on several actin-modulatory proteins, including the Rac1 and RhoA (5). Rac1 directly stimulates actin depolymerization, and β cell–specific deletion of Rac1 inhibits F-actin disassembly and impairs glucose tolerance and GSIS in mice (30). By contrast, RhoA regulates actin polymerization. The RhoA/ROCK pathway is responsible for the stabilization of actin cytoskeleton and inhibition of insulin secretion under physiological conditions (31).

To further validate the role of GIV in regulating cytoskeletal remodeling, we performed low-glucose and high-glucose treatments on β cells infected with control and GIV shRNA. Using GST-PAKI-PBD pull-down assay and GST-Rhotekin-RBD pull-down assay, we observed that high-glucose treatment significantly increased GTP-bound Rac1 (active Rac1) levels and decreased GTP-bound RhoA (active RhoA) levels in β cells (Fig. 5, *F*–*H*). However, after the downregulation of GIV, elevated glucose treatment could not activate GTP-bound Rac1 levels, while the GTP-bound RhoA levels were maintained at a low glucose level (Fig. 5, *F*–*H*). Since the total levels of Rac1 and RhoA were unchanged (Fig. 5, *F*, *I* and *J*), this result suggests that GIV is required for glucose-induced Rac1 activation and inhibition of RhoA activity. These results indicate that GIV is involved in regulating the transformation of RhoA activity into Rac1 activity in β cells, thus mediating the remodeling of the cytoskeleton.

### GIV mediates actin cytoskeleton remodeling in insulin granule exocytosis

**Figure 6:**
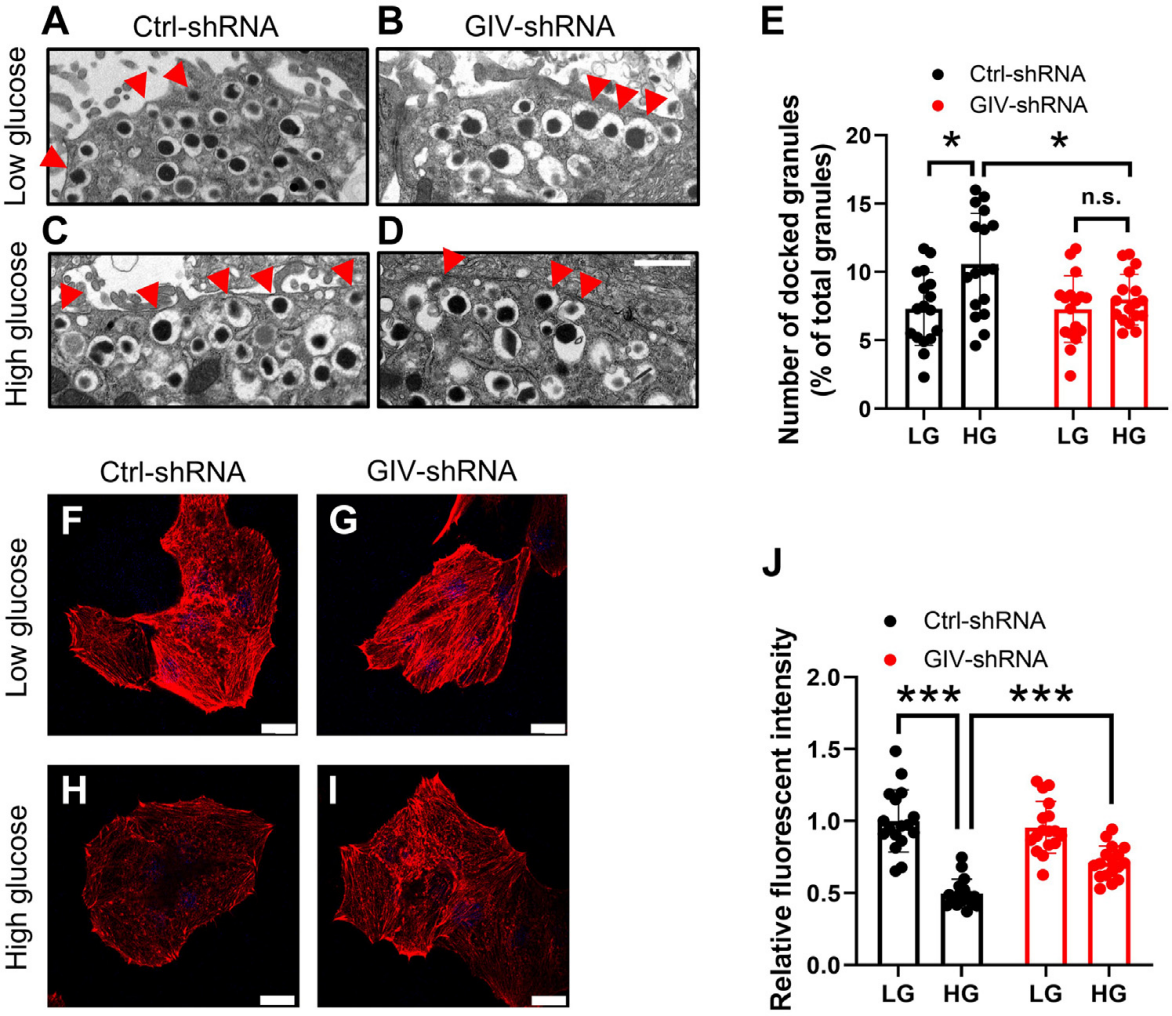
**GIV signaling promotes insulin granule exocytosis via F-Actin remodeling.***A*–*D*, electron micrographs of β cells from GIV knockdown islets with 2.8 mM low-glucose- or 16.7 mM high-glucose-containing Krebs–Ringer bicarbonate buffer incubation. Insulin granules whose centers were located within 200 nm of the plasma membrane were categorized as docked granules and were indicated by *red arrowheads*. The scale bar represents 1 μm. *E*, the number of docked granules was calculated from 18 individual β cells by Student’s *t* test. ∗*p* < 0.05; n.s. means not significant. *F*–*I*, shRNA-treated mouse pancreatic β cells were incubated with 2.8 mM glucose and 16.7 mM glucose for 30 min, then cells were fixed and stained with phalloidin. The scale bar represents 20 μm. *J*, F-actin patterns in MIN6 cells were quantified as the fluorescent intensity of phalloidin by Student’s *t* test (n = 17). ∗∗∗*p* < 0.001.

We employed ultrastructural analyses to further probe the requirement for GIV activity in insulin secretion. Electron microscopy revealed an increase in insulin granule localization at the β cell plasma membrane, in response to the high-glucose challenge, compared with the low-glucose state in normal murine islets (Fig. 6, *A*, *C* and *E*). However, GIV knockdown islets exhibited suppressed glucose-induced recruitment of insulin granules to the cell periphery but did not affect insulin docking under basal conditions (Fig. 6, *B*, *D* and *E*). GIV knockdown β cells had fewer docked insulin granules (quantified as granules within 200 nm of the plasma membrane) in the presence of high glucose. Since insulin content was unaffected by GIV-shRNA treatment (see Fig. 2*A*), the reduction in surface-localized insulin granules is not due to the defects in insulin biogenesis. These results suggest that GIV activity is crucial for glucose-dependent insulin granule positioning at the β-cell surface.

Actin cytoskeleton rearrangement is a critical acute determinant of GSIS (5). Actin microfilaments are organized as a dense meshwork beneath the β-cell plasma membrane that restricts insulin granule access to the docking and fusion machinery (32). Glucose stimulation rapidly promotes filamentous actin (F-actin) remodeling to mobilize insulin granules to the cell periphery. However, how glucose stimulation regulates actin reorganization remains unclear. Therefore, we intended to explore whether GIV signaling promotes actin rearrangements in β cells, a prerequisite step in insulin granule positioning at the plasma membrane. Isolated Ctrl-shRNA and GIV-shRNA-treated β cells were stimulated with either low or high glucose, and F-actin was visualized using Alexa 546–labeled phalloidin. Under basal conditions, both Ctrl-shRNA and GIV-shRNA-treated β cells demonstrated a thick F-actin pattern (Fig. 6, *F* and *G*). However, high-glucose treatment elicited a striking reduction in F-actin (Fig. 6, *H* and *J*), consistent with previous reports. By contrast, the glucose-induced dissolution of F-actin was prevented by GIV-shRNA-mediated silencing of GIV signaling (Fig. 6, *I* and *J*). Therefore, GIV activity is required for glucose-triggered actin reorganization in β cells.

### Rescue of GIV restores the impaired glucose-stimulated insulin secretion from db/db islets

**Figure 7:**
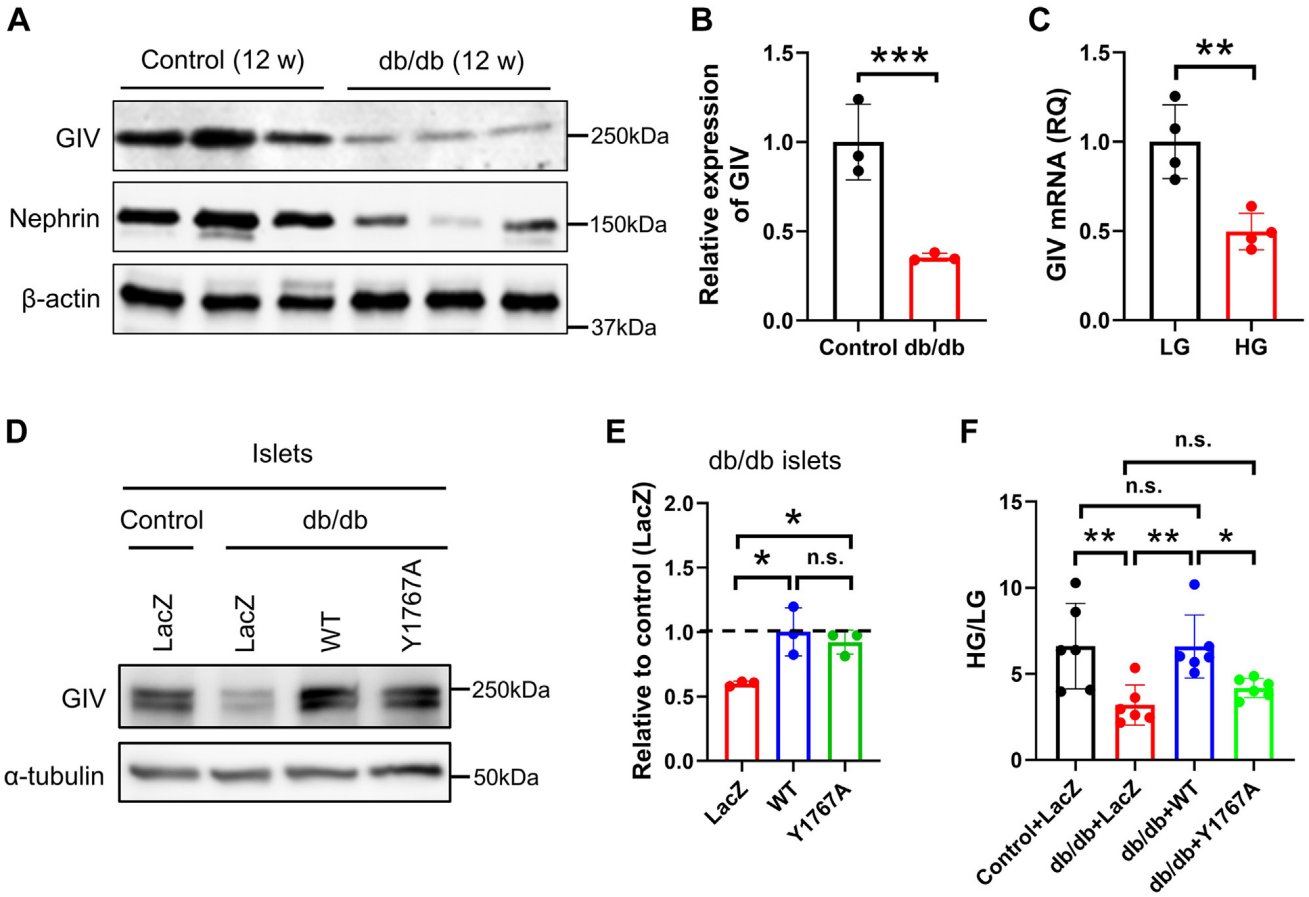
**GIV restored impaired glucose-induced insulin secretion.***A*, protein expression level of GIV and Nephrin in islets from type 2 diabetic (db/db) mice and nondiabetic (*control*) mice. *B*, the band intensity of each protein from db/db islets was normalized by those of control islets (n = 3). *C*, MIN6 cells “starved” in 2.8 mM glucose for 7 days, followed by incubation with either 2.8 mM low glucose (LG) or 25 mM high glucose (HG) for 10 days. Relative quantification (RQ) of GIV mRNA expression was detected by quantitative PCR (n = 4). *D*, the control and db/db islets were infected with adenoviruses encoding control LacZ, wildtype, or GIV-Y1767A mutant. After a 1-h infection, the islets were rinsed and incubated for 48 h at 37 °C. The protein level of GIV in db/db islets was adjusted to that of endogenous GIV in control islets by immunoblotting with the anti-GIV antibody. *E*, the protein levels of GIV normalized by α-tubulin were measured by densitometry. The *dashed line* indicated the endogenous expression level of GIV in control islets (n = 3). *F*, the control and db/db islets were infected with adenoviruses with the condition described in *D*. The islets were preincubated in 2.8 mM glucose-containing Krebs–Ringer bicarbonate buffer for 1 h and were incubated in LG or HG buffer for 1 h. Insulin secretion was shown as the ratio of HG and LG (n = 6). The statistical significance of differences between means was assessed by the Student’s *t* test for *B* and *C* and by one-way ANOVA with a Tukey’s test for *E* and *F*. ∗*p* < 0.05, ∗∗*p* < 0.01; ∗∗∗*p* < 0.001; n.s. means not significant.

We studied GIV expression in murine islets isolated from db/db (type 2 diabetes model) and control mice. The protein expression level of GIV in db/db islets was remarkably lower than in the control islets (Fig. 7, *A* and *B*), which indicated that the reduction of GIV is associated with the glucolipotoxicity of the islet. To understand whether high glucose regulated GIV expression as observed in the diabetic animal model, we studied the GIV expression of wildtype islets after chronic exposure to glucose. The result showed that chronic exposure to 20 mmol/l glucose significantly decreased the mRNA level of GIV of murine islet cells (Fig. 7*C*), consistent with the data from db/db islets. Next, we performed rescue experiments by adenovirus-mediated expression of wildtype and GIV-Y1767A mutant in db/db islets to match the endogenous level of GIV protein in control islets (Fig. 7, *D* and *E*). The GSIS results revealed that the db/db islets exhibited markedly reduced insulin secretion than the wildtype islets, whereas wildtype GIV significantly restored GSIS to the level found in wildtype islets expressing the control LacZ protein. In contrast, the GIV-Y1767A mutant could not rescue the impaired GSIS in db/db islets (Fig. 7*F*). These findings demonstrate the potent role of GIV in restoring impaired β-cell function in diabetic islets.

## Discussion

This study unveiled that GIV affects the function of pancreatic β cells and promotes insulin secretion in response to glucose. This function of GIV is independent of its well-known and described role in kidney podocytes, where it regulates the structure and function of the glomerular slit diaphragm (24). This study, for the first time, demonstrates that GIV not only is a master regulator of cell–cell adhesion in podocytes but also affects insulin signaling in islet β cells by the regulatory role of GIV regulating Nephrin phosphorylation and endocytosis. Generally, Nephrin is distributed on the plasma membrane of islet cells. It anchors stress fibers to form a mesh-like barrier that prevents insulin vesicles from contacting and fusing with the plasma membrane (13). Previous studies have revealed that Nephrin phosphorylation and endocytosis are core signaling events governing GSIS (11, 14). Nevertheless, the detailed mechanism of the glucose-induced Nephrin endocytosis for insulin secretion remains unclear.

**Figure 8:**
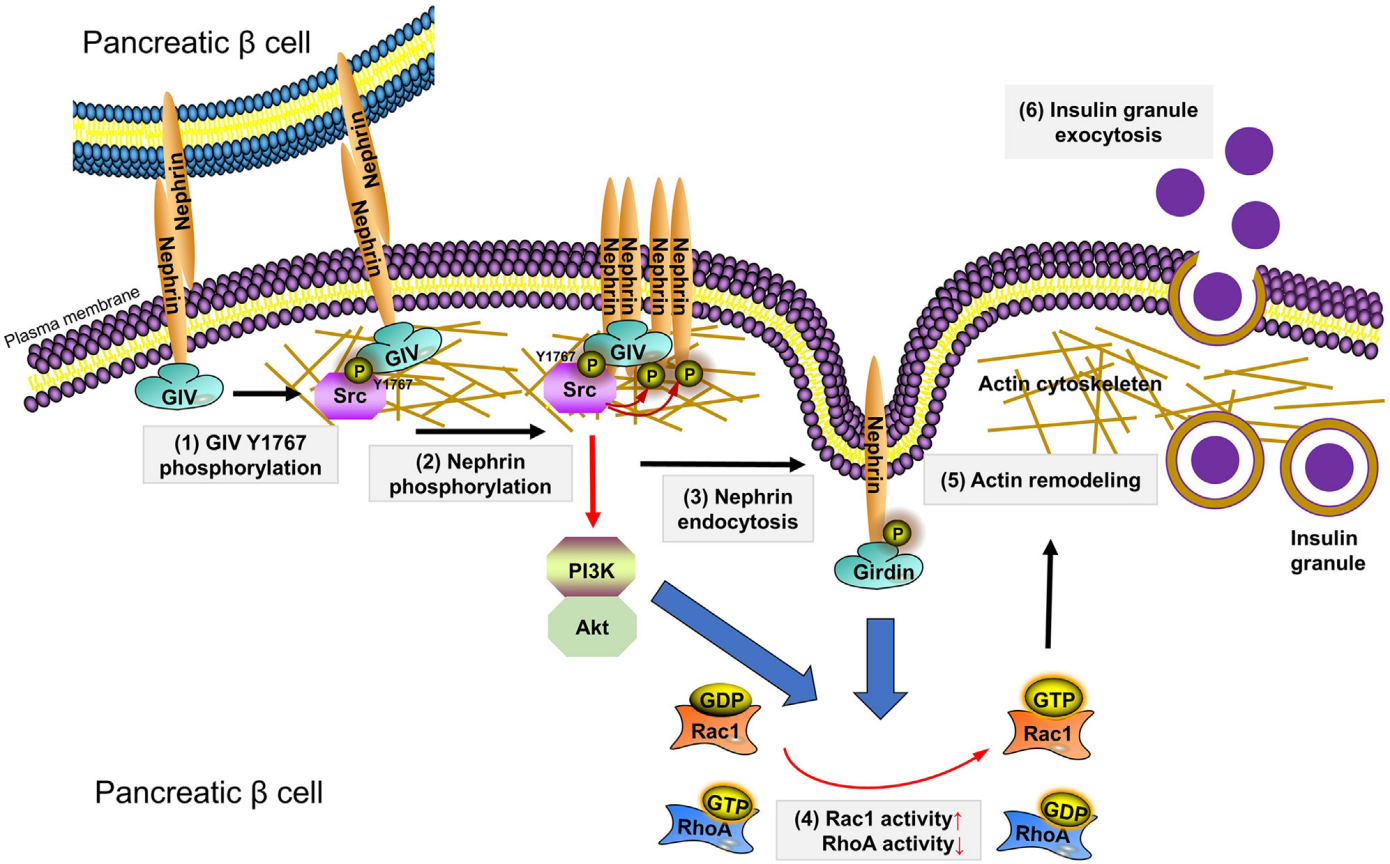
**GIV acutely promotes Nephrin-dependent glucose-stimulated insulin secretion via actin cytoskeleton reorganization in β cells.** (1) Src kinase induced GIV-Y1767 phosphorylation in response to elevated glucose. (2) GIV-Y1767 phosphorylation recruited Src kinase to Nephrin for phosphorylating Nephrin. (3) Nephrin phosphorylation induced Nephrin endocytosis pathway. (4) Cytoplasm Nephrin from endocytosis and PI3K/Akt signaling regulated activities of actin-modulatory protein Rac1 and RhoA, to (5) permeabilize a peripheral F-actin barrier, and (6) promoted insulin granule exocytosis.

In this study, we found that GIV functions as a binding partner of Nephrin to colocalize on the plasma membrane in β cells. Upon high-glucose stimulation, GIV is phosphorylated at Y1767 by the activated Src kinase. As an intermediator, the phosphorylated GIV recruited Src kinase to phosphorylate Nephrin since both phosphorylation and Src binding of Nephrin was disrupted after the reduction of GIV. We also found glucose-induced Y1767 phosphorylation of GIV, followed by PI3K/Akt signaling stimulation. Our rescue experiment data validated that deficient phosphorylation of GIV at Y1767 could also effectively inhibit insulin secretion of MIN6 cells and in islets (Fig. 4*G*). Furthermore, we found that Src kinase, but not Fyn and Lck, as well as Lyn kinase, is required for interactions of GIV with Nephrin in murine islet β cells. Accordingly, we propose the following scenario demonstrating the role of phosphorylation of GIV at Y1767 during glucose-stimulated insulin secretion, and the recruitment of Src to interact with Nephrin is essential for Nephrin endocytosis and phosphorylation. Subsequently, the activated GIV functions in triggering the PI3K-dependent Akt phosphorylation, which facilitates the GSIS by activating Rac1 and RhoA to reorganize the actin cytoskeleton in islet β cells (Fig. 8).

In this proposed model, we argue that GIV-mediated phosphorylation of Nephrin causes the aggregation of the regulator of endocytosis, such as Dynamin, to regulate its endocytosis (11). Consequently, the endocytosis of Nephrin activates the function of the essential regulator of the cytoskeleton, including the Rac1 and RhoA, which reduce the stress fibers that act as a barrier by actin remodeling (30, 31). Therefore, insulin vesicles can fuse with the cell membrane and then release insulin (3). We also found that the Src kinase inhibitor significantly retarded high-glucose-induced phosphorylation of the Nephrin–GIV complex, blocking Nephrin endocytosis and insulin release. The role of GIV in recruiting the Src kinase to bind and phosphorylate Nephrin/GIV complexes has also been identified using the loss of function experiment by the knockdown of GIV in pancreatic islet β cells. Therefore, we propose that GIV mediates the recruitment of nonreceptor kinase Src to amplify the tyrosine phosphorylation of Neprhin, which plays a role in the effect of sensitive glucose concentration.

Recently, GIV has been found to be localized on E-cadherin-mediated cell–cell junctions, contributing to Wnt/β-catenin activation to reorganize the cytoskeleton and migration of mammalian cells (33). On the other hand, the multifunctional GIV protein has been well recognized as an intracellular signaling mediator to propagate various signals across the plasma membrane (17). In this study, we manipulated GIV expression in insulinoma MIN6 cells and dissociated murine islets and established the novel function of GIV in GSIS. Upon insulin stimulation or high-glucose condition, the total level and Y1767-phosphorylation of GIV were significantly increased. Our results demonstrate that the increased Y1767-phosphorylation of GIV positively correlates with the Src kinase activation, as shown that the Src kinase inhibitor, saracatinib, could effectively abolish the GIV phosphorylation (Fig. 4*C*). Using the adenovirus containing wildtype GIV and its mutant of Y1767A, we verified the essential role of Y1767 phosphorylation of GIV in GSIS *via* the rescue experiments in both GIV-depleted MIN6 cells and the islets from type 2 diabetic db/db mice (Figs 4*G* and 7*F*). Notably, GIV is known to act as a nonreceptor GEF to modulate trimeric G protein activity to affect Akt signaling (19, 20), which might also contribute to the regulatory machinery of GSIS. However, this possible mechanism remains to be explored in the future study, and it would be highly intriguing to further investigate whether GIV-mediated G protein activation plays a role in GSIS and how Y1767-phosphorylation structurally affects the GEF function of GIV for signal propagation. Moreover, the increased expression level of GIV may be the consequent events through a feedback mechanism upon high glucose, which led to the dissociation of the membrane-associated Nephrin from connected β cells to initiate endocytosis, thereby increasing the fraction of the Triton X-100 soluble complex of GIV/Nephrin in the lysates (Fig. 3*C*).

Our research reveals that F-actin remodeling is induced upon the GIV-mediated endocytosis of Nephrin after glucose load in β cells. The role of GIV in cytoskeleton regulation has been reported that it can directly bind to actin filaments and stabilize F-actin (15). Upon shRNA-mediated knockdown of GIV, stress fibers were disrupted to alter the cell morphology, suggesting the role of GIV in cytoskeleton-mediated cellular processes, such as insulin secretion. Our results showed that downregulation of GIV strongly impaired the F-actin depolymerization after glucose stimulation, thereby inhibiting subsequent insulin granule exocytosis in β cells. In comparison, GIV depletion did not affect the KCl-stimulated insulin secretion in MIN6 cells (Fig. 2*D*). Combining the electron microscopic observation, our work validates the function of GIV in properly localizing the insulin granules on the β-cell surface *via* cytoskeleton remodeling in the second phase of GSIS, however, not the granule fusion with the plasma membrane in the first phase of insulin secretion.

Another interesting finding of this study is that GIV expression is decreased in islets from diabetic (db/db) mice compared with age-matched control (db/m) mice, which was different from what was previously reported in the kidney (24). Consistent with these ideas, prolonged exposure to high glucose led to a downregulation of GIV expression in MIN6 cells, while acute exposure to high glucose increased GIV mRNA expression. Although the relevance of an *in vitro* model of acute and prolonged glucose exposure in insulinoma cells remains to be established, the opposite effect of chronic glucose exposure on GIV gene expression is contrary to what has been described in kidney cells (24), where chronic glucose exposure has been widely accepted as a model of glucotoxicity (13). One possible interpretation is that GIV controls the endocytosis of Nephrin to facilitate insulin secretion, which may be impaired in diabetes because of the downregulation of GIV in pancreatic β cells. Further investigation would be highly desirable to define the precise mechanism whereby GIV augments insulin secretion.

In conclusion, we have established that GIV is an important regulator of glucose-stimulated insulin secretion in pancreatic β cells. Our proposed working model suggests that GIV mediates the high glucose sensing of Nephrin and insulin secretion *via* the recruiting of Src kinase to trigger the tyrosine phosphorylation of Nephrin and GIV. The tyrosine phosphorylation of Nephrin initiates the Nephrin endocytosis, thereby affecting subsequent insulin exocytosis by PI3K/Akt-mediated actin remodeling. Therefore, this study identifies a novel signaling machinery regulating glucose-stimulated insulin secretion, which might be a potential target for the therapy of diabetic mellitus. Selective intervention to restore GIV expression or function may delay the need for insulin therapy in type 2 diabetes. Further exploration is crucial to characterize the role of GIV in regulating blood glucose homeostasis for a long-term study *in vivo*.

## Experimental procedures

### Cell culture and islet culture

All cells were cultured in a humidified incubator with 95% air and 5% CO_2_ at 37 °C. MIN6 cells (34) were cultured in Dulbecco's modified Eagle's medium containing 15% fetal bovine serum supplemented with 50 μM 2-mercaptoethanol. INS1 832/13 cells (35) were cultured in RPMI1640 containing 10% fetal bovine serum supplemented with 1 mM L-glutamine, 1 mM Hepes, 1 mM sodium pyruvate, and 50 μM 2-mercaptoethanol. HEK293A cells (Invitrogen) were cultured in Dulbecco’s modified Eagle’s medium containing 10% fetal bovine serum supplemented with 1 mM L-glutamine. Pancreatic islets were isolated from mice sacrificed by cervical dislocation through injection of 500 units/ml collagenase solution (type XI; Sigma) into the pancreatic duct, followed by mild shaking digestion at 37 °C for 20 min, and isolated islets were manually selected under a dissecting microscope, as described elsewhere. Isolated islets were cultured overnight in RPMI 1640 medium containing 10% fetal bovine serum, 100 units/ml penicillin-streptomycin. After overnight recovery, islets were treated with 0.05% trypsin in PBS at 37 °C for 5 min, fully dispersed to primary β cells. The C57BL/6J mice, type 2 diabetic db/db mice, and nondiabetic control mice were purchased from Jiangsu Gempharmatech Laboratories. The mice were kept at constant temperature and humidity, with a 12-h light and dark cycle, and fed a regular unrestricted diet. Only male mice were used in our experiments. All animal experiments were performed in accordance with the national ethical guidelines implemented by our Institutional Animal Care and Use Committee and approved by the Ethical Review Committee of the Institute of Zoology, Capital Medical University, China.

### Construction of the lentivirus and adenovirus vectors

Short hairpin RNA (shRNA) with nontargeting (CCTAAGGTTAAGTCGCCCTCG) or mouse Girdin targeting CDS region (CAGTCGATTCATCACCACCTA) was cloned into the PLKO.1 puro vector (Invitrogen). For the downregulation of GIV, MIN6 cells and islets were infected with GIV shRNA lentivirus. After 48-h infection, the cells or the islets were used for protein and functional assay. For the rescue experiment, shRNA with mouse Girdin targeting 3′-UTR sequence (GCAACTATAGGAACTATTAAA) was cloned into the PLKO.1 puro vector (Invitrogen). Full-length mouse GIV cDNA was cloned from MIN6 cells. Wildtype GIV was performed using the following primers: F1 5′-GGTCGACTCTAGAGGATCCAAGCCACCATGGAGAACGAAATCTTCACTCCCC-3′ and R1 5′-AATGACACGGTCTGCCTCTGC-3′ as well as F2 5′- CTCAGATTCTTGCACTGCAGAGG-3′ and R2 5′-TCCTTGTAGTCCATACCGGTAATACAACCATATTCATACCAAACAATTTGGCT-3′; site-directed mutagenesis of GIV-Y1767A was generated from wildtype of GIV as a template and performed using the following primers: forward 5′-AAGACTGAAGATGCCGCCACCAT-3′ and reverse 5′- AGAGCTGATGGTGGCGGCAT-3′. To generate recombinant adenoviruses, they were inserted into pENTR-3C (Invitrogen) and were transferred into pAd/CMV by LR Clonase recombination (Invitrogen). To express the exogenous protein, MIN6 and islets were infected with adenoviruses encoding GIV. For the rescue experiment of GIV downregulated MIN6 cells, MIN6 cells were first infected with lentivirus encoding GIV-shRNA targeting mouse 3′-UTR sequence and nontargeting sequence for 48 h, then they were infected with lentivirus encoding GIV-shRNA again followed with adenovirus encoding LacZ, wildtype GIV, or Y1767A mutant GIV for another 48 h. For the rescue experiment of db/db islets, db/db and control islets were infected with adenovirus encoding LacZ, wildtype GIV, or Y1767A mutant GIV for 48 h before being used for protein or functional assays.

### Immunoblotting and immunoprecipitation

The sources of antibodies and their concentrations used are listed in Table S1. Cell lysate proteins, separated by gel electrophoresis, were transferred onto an Immobilon-P membrane (Millipore) and visualized by enhanced chemiluminescence (GE Healthcare Biosciences). Cells were lysed in lysis buffer containing 20 mM Tris-HCl pH 7.5, 150 mM NaCl, 1 mM MgCl_2_, 1% Triton X-100, 1 mM PMSF, and complete protease inhibitor cocktail (Roche). The lysates were cleared by centrifugation at 14,000 rpm for 15 min at 4 °C. The supernatants were subjected to immunoprecipitation with primary antibody and Protein G-Sepharose 4F (GE Healthcare Bioscience). After being washed five times with wash buffer containing 20 mM Tris-HCl pH 7.5, 150 mM NaCl, 10% Glycerol, 0.1% TritonX-100, the immunoprecipitates were subjected to SDS-PAGE and then transferred to a polyvinylidene difluoride membrane. The membrane was blocked with TBST (TBS plus 0.1% Tween-20) containing 0.5% nonfat dried milk powder and then incubated overnight at room temperature with the primary antibody. It was then washed three times with TBST and incubated for 1 h at room temperature with a 5000× dilution of horseradish peroxidase–conjugated secondary antibody (GE Healthcare Bioscience) in TBST containing 0.5% nonfat dried milk powder, and was washed five times. Immunoreactive signals were then detected using ECL prime and an LAS-4000 chemiluminescence detection system (GE Healthcare Bioscience).

### Glucose-stimulated insulin secretion assay

MIN6 cells plated on 24-well plates were cultured for 24 h. The cells were incubated in low-glucose Krebs–Ringer bicarbonate buffer (120 mM NaCl, 5 mM KCl, 24 mM NaHCO_3_, 1 mM MgCl_2_, 2 mM CaCl_2_, 15 mM Hepes pH 7.4, 0.1% bovine serum albumin, 2.8 mM glucose) for 1 h followed by the same buffer or a high-glucose buffer containing 16.7 mM glucose for another 1 h. For mouse islets, batch assay and perfusion assay were performed as described (36). Insulin levels were measured with a mouse insulin ELISA kit (Mercodia 10-1247-01) with an Infinite 200 Pro Reader (TECAN).

### Immunofluorescence and microscopy

Murine pancreatic frozen sections and pancreatic β cells from C57BL/6N were fixed with 3% paraformaldehyde in phosphate-buffered saline (PBS) for 30 min and permeabilized with 0.1% Triton X-100 in PBS for 30 min. The cells were then treated with 50 mM NH_4_Cl-PBS for 10 min at room temperature and blocked with PBS containing 1% bovine serum albumin for 15 min. The coverslips were incubated with primary antibody overnight, washed three times with PBS, and incubated with Alexa Fluor 488– or Alexa Fluor 568–conjugated secondary antibody (Invitrogen; 1:500 dilution) for 60 min. Samples were washed five times and mounted using SlowFade Gold (Invitrogen). The microscopic images were obtained with an A1 (Nikon) confocal laser scanning microscope equipped with a 100× oil immersion objective lens (1.49 NA) and NIS elements. The images were adjusted using NIS elements and ImageJ software.

### RNA isolation and expression analyses

RNA was extracted using Trizol Reagent (Invitrogen) according to the manufacturer's instructions. Total RNA (1 μg) was reverse transcribed using oligo-(dT)12–18 primer and Superscript III (Invitrogen). Quantitative PCR was performed on a LightCycler 480 Real-Time PCR System (Roche) using SYBR Green I Master Mix reagent (Roche) with the primers. All reactions were run in triplicate. The relative mRNA expression level was calculated and normalized against Rplp0/36B4 mRNA expression. The primers for mouse genes were as follows: GIV forward primer 5′-GTGATCTCTACTGCTGAAGG-3′ and reverse primer 5′-TGTTGCTCCCTAGACCTGCT-3′, Rplp0/36B4 forward primer 5′-GGCCCTGCACTCTCGCTTTC-3′ and reverse primer 5′-TGCCAGGACGCGCTTGT-3′.

### Electron microscopic analysis of granule distribution

Isolated islets were incubated in low-glucose (2.8 mM glucose) Krebs–Ringer buffer at 37 °C for 1 h followed with high-glucose (16.7 mM glucose) Krebs–Ringer buffer for another 1 h. They were fixed by immersion with 2% paraformaldehyde, 2% glutaraldehyde/0.2% picric acid in 0.1 M cacodylate buffer, pH 7.4, for 1.5 h at room temperature and embedded into 1% agarose. They were then postfixed, embedded in plastic resin, and sectioned. Ultrathin sections (80 nm) were cut with microtome (Leica EM UC6) and analyzed under a transmission electron microscope (FEI Tecnai Spirit 120 kV). Micrographs were randomly taken at ×4000 magnification from 18 individual β cells from three mice for each genotype. The distance from the granule center to the plasma membrane was measured as described elsewhere (37).

### Cell-surface biotinylation assay

Start with cultured cells cooled to 4 °C. Place them on ice to maintain the temperature that is restrictive to endocytosis. Next, the membrane-impermeable sulfo-NHS-SS-biotin reagent is added, and cells are incubated in the dark for approximately 30 min. This allows sufficient time for biotin labels to covalently attach to the surface proteins. Cells are then removed from the ice and incubated at 37 °C for approximately 30 min. At this temperature, biotinylated surface proteins are endocytosed. Following incubation, the cells are cooled to 4 °C and a hydrophilic reducing agent like L-glutathione is added. This reacts with disulfide bonds and releases the biotin groups from labeled, nonendocytic proteins. Next, cells are lysed by centrifugation, thus breaking cell membranes and exposing biotinylated proteins. Following this, lysates are added to streptavidin-coated beads, and biotinylated proteins are allowed to bind. Beads are washed with cold PBS and eluted with a buffer containing detergents and reducing agents. These reagents denature bound proteins off beads and enable their recovery in the eluate. Proteins in the eluate are separated based on their molecular mass by gel electrophoresis. Lastly, Western blotting was carried out and probing the blot with protein-specific antibodies allowed the visualization of the target protein. The percentage of endocytosed protein can be quantified from the resulting band densities.

### Statistical analysis

Data are presented as means ± SD values and compared by Student’s *t* test and one-way ANOVA with a Tukey’s test. A *p* value of <0.05 was considered significant.

## Data availability

All data are contained within the article.

## Supporting information

This article contains supporting information.

## Conflict of interest

The authors declare that they have no conflicts of interest with the contents of this article.
