# Supplementary material for: The signaling protein GIV/Girdin mediates the Nephrin-dependent insulin secretion of pancreatic islet β cells in response to high glucose
Source: J Biol Chem. 2023 Feb 21;299(4):103045. doi: 10.1016/j.jbc.2023.103045 (PMC10040812; doi:10.1016/j.jbc.2023.103045)
Supplement: Supporting Figures S1–S4 and Table S1 [file mmc1.docx]

**Supporting Information for:**

**The Signaling Protein GIV/Girdin Mediates the Nephrin-dependent Insulin Secretion of Pancreatic Islet β Cells in Response to High Glucose**

Hao Wang^1,5,🖂^, Ying-Chao Yuan^1,5^, Cong Chang^2,4,5^, Tetsuro Izumi^3^, Hong-Hui Wang^2,🖂^ and Jin-Kui Yang^1,🖂^

^1^Beijing Key Laboratory of Diabetes Research and Care, Beijing Diabetes Institute, Beijing Tongren Hospital, Capital Medical University, Beijing 100730, China.

^2^College of Biology, Hunan University, Changsha 410082, Hunan, China.

^3^Laboratory of Molecular Endocrinology and Metabolism, Department of Molecular Medicine, Institute for Molecular and Cellular Regulation, Gunma University, Maebashi 3718512, Gunma, Japan.

^4^Hunan Food and Drug Vocational College, Changsha 410208, Hunan, China.

^5^These authors contributed equally: Hao Wang, Ying-Chao Yuan, Cong Chang

^🖂^Correspondence: Hao Wang (hwang2002@126.com) or Hong-Hui Wang (wanghonghui@hnu.edu.cn) or Jin-Kui Yang ([jkyang@ccmu.edu.cn](mailto:jkyang@ccmu.edu.cn))

**This file includes:**

Supplementary Table 1 for sources of commercial reagents and the concentrations of the antibodies used for immunofluorescence (IF), immunoblotting (IB), or immunoprecipitation (IP)

Supplementary Figure 1

Supplementary Figure 2

Supplementary Figure 3

Supplementary Figure 4

**Supplementary Table 1. The sources of commercial reagents and the concentrations of the antibodies used for immunofluorescence (IF), immunoblotting (IB), or immunoprecipitation (IP)**

| **Antibody** | **Clone** | **Vendor** | **Catalog number** | **RRID** | **Dilution** | **Applica**  **-tion** |
| --- | --- | --- | --- | --- | --- | --- |
| anti-GIV | Rabbit polyclonal | gift from Hunan University |  |  | 1/100 | IF |
| GIV | Goat | R & D Systems  Minneapolis, USA | AF5345 | AB_2259812 | 1/1000 | IB |
| pGIV (Y1764) | Rabbit polyclonal | ECM Biosciences | GP5801 |  | 1/1000 | IB |
| Nephrin | Rabbit polyclonal | Abcam, Cambridge, UK | ab136894 | AB_2894886 | 1/1000 | IB |
| pNephrin (Y1176+Y1193) | Rabbit polyclonal | Abcam, Cambridge, UK | ab80299 |  | 1/1000 | IB |
| HA (3F10) | Rat monoclonal | Roche Diagnostics GmbH, Mannheim, Germany | 11867423001 | AB_390918 | 1/100 | IF |
| GAPDH (3H12) | Mouse monoclonal | MBL, Nagoya, Japan | M171-3 | AB_10597731 | 1/1000 | IB |
| FLAG | Rabbit polyclonal | Sigma-Aldrich, St.Louis, MO, USA | F7425 | AB_439687 | WB 1/5000; | IB |
| β-actin | Mouse monoclonal | Sigma-Aldrich, St.Louis, MO, USA | A5316 | AB_476743 | 1/10000 | IB |
| α-tubulin | Mouse monoclonal | Sigma-Aldrich, St.Louis, MO, USA | T5168 | AB_477579 | 1/10000 | IB |
| Akt | Rabbit polyclonal | Cell Signaling Technology, Inc., MA, USA | 9272 |  | 1/1000 | IB |
| pAkt (S473) | Rabbit polyclonal | Cell Signaling Technology, Inc., MA, USA | 4060S |  | 1/1000 | IB |
| Insulin | Mouse monoclonal | Cell Signaling Technology, Inc., MA, USA | 8138S | AB_10949314 | 1/500 | IF |
| Glucagon | Mouse monoclonal | Sigma-Aldrich, St.Louis, MO, USA | S2654 | AB_259852 | 1/200 | IF |
| c-Src | Mouse monoclonal | Santa Cruz Biotechnology Inc., CA, USA | SC-5266 |  | 1/2000 | IB |
| pSrc (Y419) | Rabbit polyclonal | Abcam, Cambridge, UK | ab4816 |  | 1/1000 | IB |
| Fyn | Mouse monoclonal | Santa Cruz Biotechnology Inc., CA, USA | SC-434 |  | 1/1000 | IB |
| Lck | Rabbit polyclonal | Proteintech, China | 12477-1-AP |  | 1/2000 | IB |
| Lyn | Rabbit polyclonal | Proteintech, China | 18135-1-AP |  | 1/2000 | IB |
| Rac1 | Mouse monoclonal | Sigma-Aldrich, St.Louis, MO, USA | 05-389 |  | 1/2000 | IB |
| RhoA | Mouse monoclonal | Sigma-Aldrich, St.Louis, MO, USA | 05-778 |  | 1/2000 | IB |
| Na-K-ATPase | Rabbit Monoclonal | Sigma-Aldrich, St.Louis, MO, USA | ZRB1574 |  | 1:1000 | IB |
| Rhodamine Phalloidin |  | Invitrogen, Thermo Fisher Scientific, Waltham, MA USA | R415 |  | 1/400 | IF |
| Protein G Sepharose 4 Fast Flow |  | GE Healthcare Biosciences, Uppsala, Sweden | GE17-0618-01 |  | 40 μl/sample | IP |
| Ly294002 |  | MCE | HY-10108 |  |  |  |
| Saracatinib |  | MCE | HY-10234 |  |  |  |

Supplementary Figures


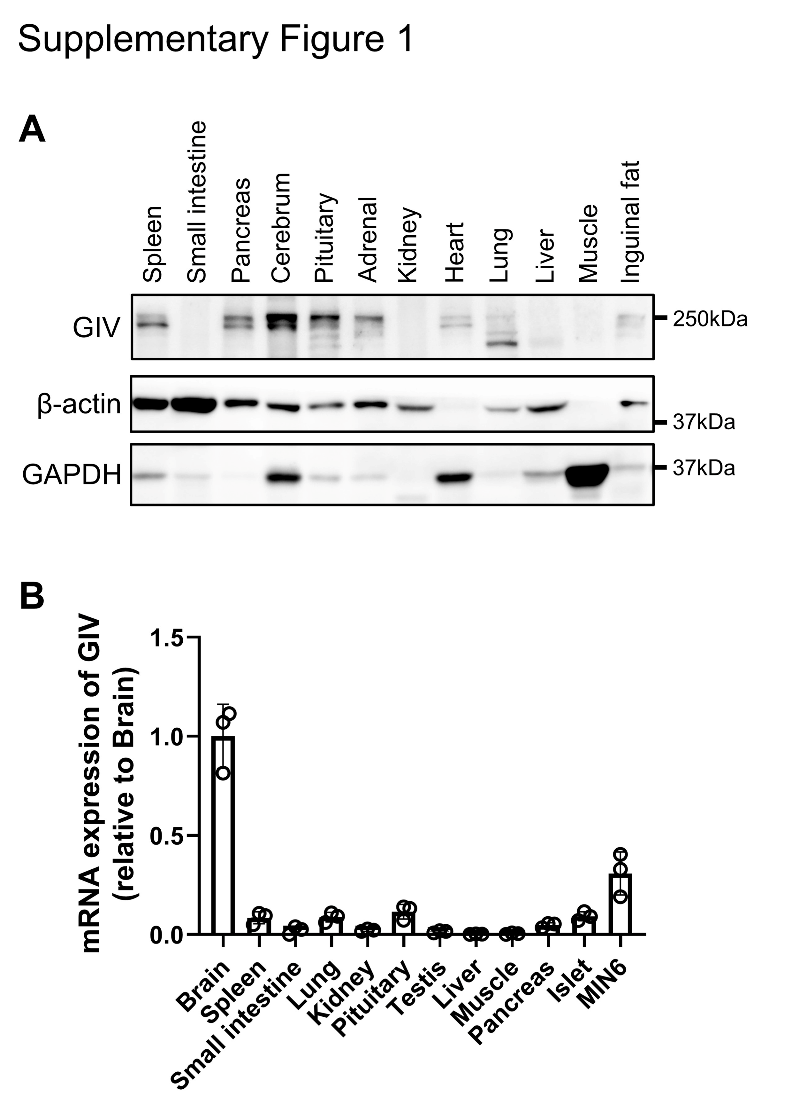


Supplementary Figure 1. Expression of GIV in different tissues.

A: The total protein lysates (20 μg) from C57BL/6J mice were analyzed by immunoblotting with anti-GIV antibody (n = 3). B: GIV mRNA expression in different tissues and cells (n = 3).


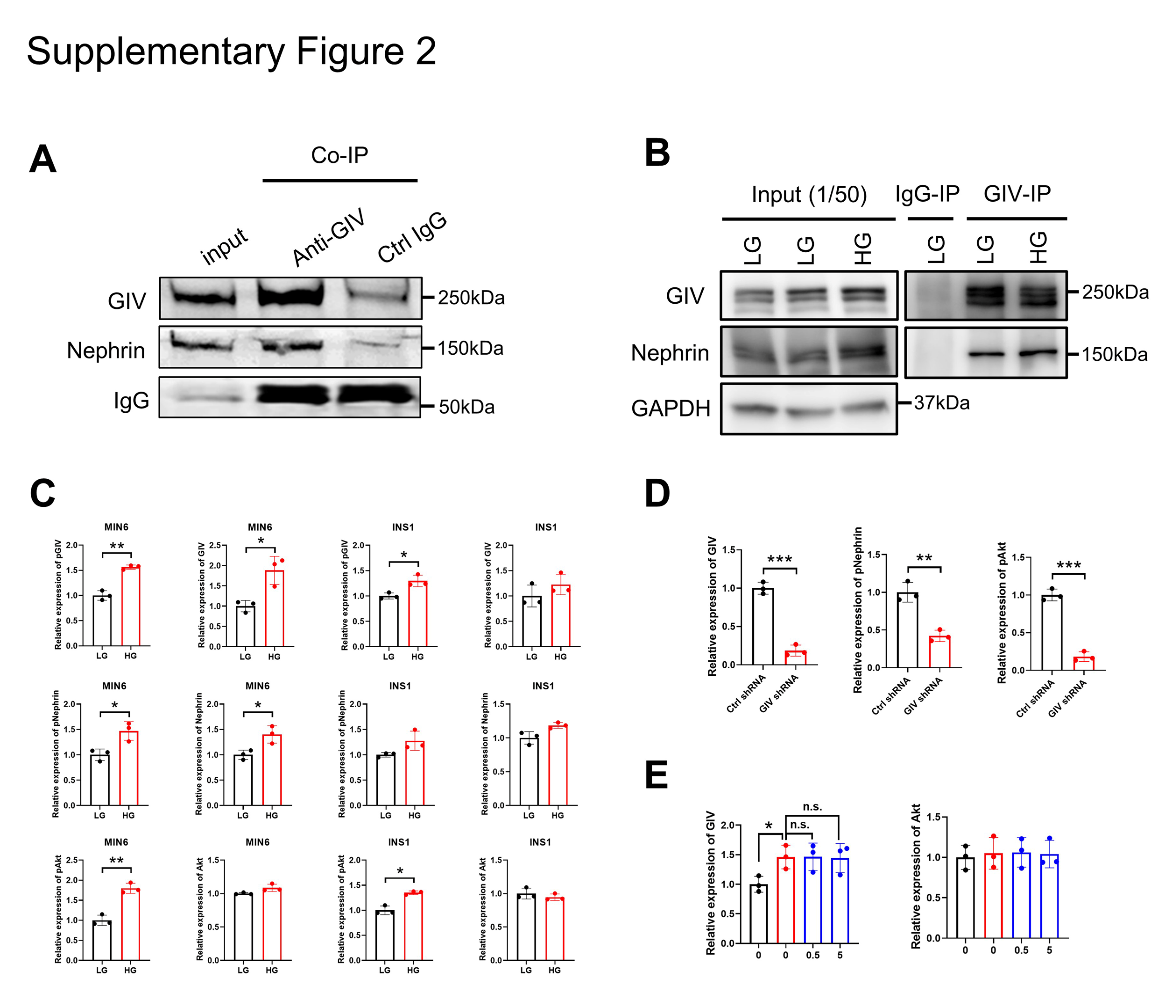


Supplementary Figure 2. Relative change of the band intensity for corresponding proteins in Figure 3.

A: Total islet protein lysates (300 mg) from wild-type mice underwent immunoprecipitation with anti-GIV antibody or control IgG. The immunoprecipitates, as well as 1:20 of the original lysates, were immunoblotted with anti-GIV and anti-Nephrin antibodies (n = 3). B: MIN6 cells were incubated in KRBB containing 2.8 mmol/L glucose for 30 min at 37°C. They were then stimulated with 2.8 mmol/L (LG) or 25 mmol/L glucose (HG) for 30 min. The cells were lysed, and the lysates underwent immunoprecipitation with control IgG and anti-GIV antibody. The immunoprecipitate, as well as 1:50 of the original lysates, were immunoblotted with the indicated antibodies (n = 3). C: The band intensity of each protein corresponding to Fig.3C was normalized by that of 2.8 mM glucose-treated cells. The statistical significance of differences between means was assessed by Student *t*-test. **p*＜0.05; ***p*＜0.01. D: The band intensity of each protein of GIV-shRNA treated cells corresponding to Fig.3D was normalized by that of Ctrl-shRNA treated cells. The statistical significance of differences between means was assessed by Student *t*-test. ***p*＜0.01; ****p*＜0.001. E: The total protein levels corresponding to Fig.3E were normalized by internal reference. The statistical significance of differences between means was assessed by one-way ANOVA with a Tukey’s test. **p*＜0.05; n.s. means not significant.


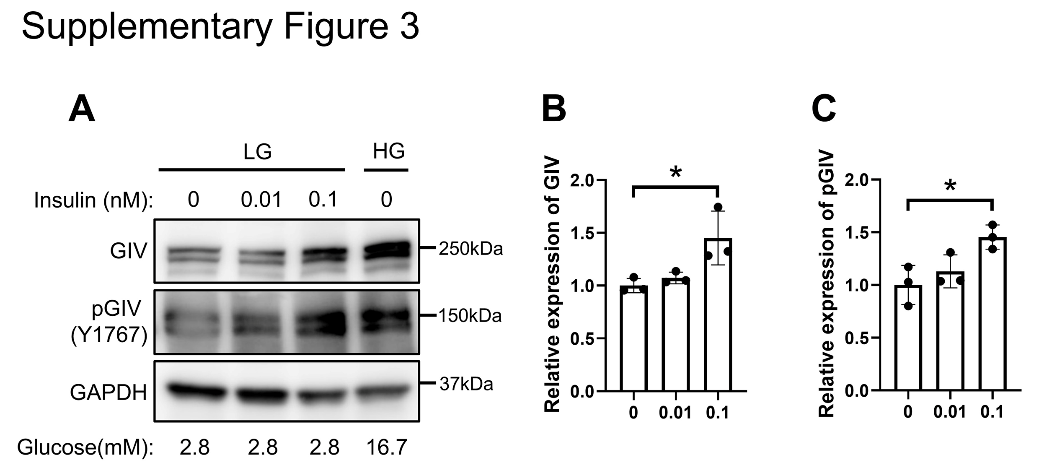


Supplementary Figure 3. Insulin induced Y1767 phosphorylation of GIV.

A: MIN6 cells were stimulated with 2.8mM or 16.7 mM glucose for 30 min in the presence or absence of insulin, 16.7 mM glucose treated was as a positive control of GIV phosphorylation (n = 3). B: The band intensity of each 2.8 mM glucose-treated protein corresponding to A was normalized by that of 0 nM insulin-treated cells. The statistical significance of differences between means was assessed by one-way ANOVA with a Tukey’s test. **p*＜0.05 versus 2.8 mM glucose with 0 nM insulin.


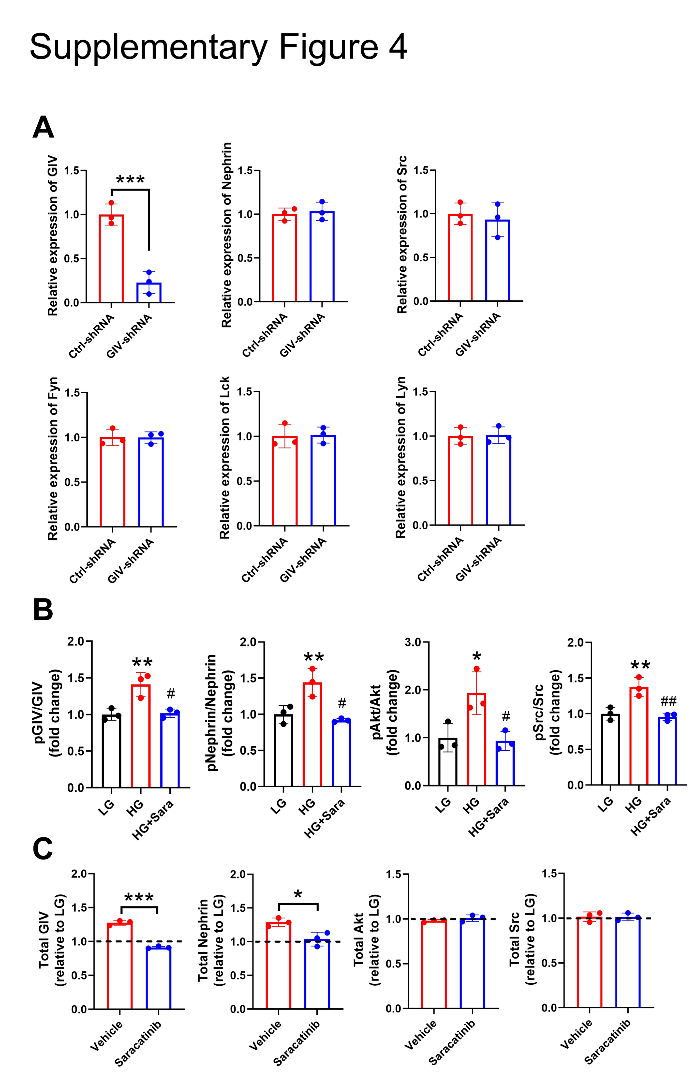


Supplementary Figure 4. Relative change of the band intensity for corresponding proteins in Figure 4.

A: Total protein expression levels in Ctrl-shRNA or GIV-shRNA treated MIN6 cells corresponding to Fig.4A were normalized by internal reference. B: The expression levels of phosphorylated proteins in high glucose plus vehicle or saracatinib treated MIN6 cells corresponding to Fig.4C were normalized by their total protein levels. The statistical significance of differences between means was assessed by One-way ANOVA with a Tukey’s test. **p*＜0.05; ***p*＜0.01 versus 2.8 mM glucose, ^#^*p*＜0.05; ^##^*p*＜0.01 versus 16.7 mM glucose with DMSO. C: Protein expression levels in high glucose plus vehicle or saracatinib treated MIN6 cells correspond to Fig.4C were normalized by internal reference. The dash line indicated the protein level in low glucose plus vehicle MIN6 cells. The statistical significance of differences between means was assessed by Student *t*-test. **p*＜0.05; ****p*＜0.001.
